# Topical use of adrenaline in different concentrations for endoscopic sinus surgery

**DOI:** 10.1016/S1808-8694(15)30791-6

**Published:** 2015-10-19

**Authors:** Krishnamurti Matos de Araujo Sarmento, Shiro Tomita, Arthur Octavio de Ávila Kós

**Affiliations:** 1 Otorhinolaryngologist. Per-oral endoscopist. Master's degree student in otorhinolaryngology, UFRJ. Coordinator of the medical residency program in otorhinolaryngology, Hospital Geral de Bonsucesso, RJ; 2 Full professor of the Otorhinolaryngology Unit, Universidade Federal do Rio de Janeiro. Head of the Otorhinolaryngology Unit, UFRJ; 3 Professor Emeritus of Otorhinolaryngology, Universidade Federal do Rio de Janeiro

**Keywords:** paranasal sinus diseases, epinephrine, hemostasis surgical, norepinephrine, nasal polyps

## Abstract

The ideal adrenaline concentration remains unknown.

**Aim:**

Compare topical adrenaline solutions in different concentrations.

**Study design:**

Prospective, double blind, randomized trial.

**Patients and methods:**

49 patients divided in 3 groups underwent endoscopic sinus surgery, using only topical solutions of adrenaline in different concentrations (1:2,000, 1:10,000 and 1:50,000). We compared the duration of surgery, intra-operative bleeding, plasmatic levels of catecholamines, hemodynamic parameters and changes in heart rhythm.

**Results:**

Surgery time was shorter in the group using adrenaline 1:2,000, which also showed less bleeding in all evaluations (objective and subjective - p < 0.0001). Plasmatic levels of epinephrine rose in all groups, more sharply in the 1:2,000 group. There was a trend towards elevation of blood pressure in the groups using adrenaline 1:2,000 and 1:10,000, with a greater occurrence of hypertensive peaks.

**Discussion:**

We found a very significance bleeding difference favoring the 1:2,000. The blood pressure elevation in the 1:2,000 and 1:10,000 groups was progressive but very slow throughout the procedure, which could be associated with the anesthesia technique.

**Conclusion:**

We favor the use of topical adrenalin 1:2,000 due to a clear superiority in hemostasis. Further investigation is needed to corroborate our findings.

## INTRODUCTION

A limiting factor in nose surgery has been the difficulty in manipulating surgical instruments in the nasal fossae and attaining a good view of the operating field. The main hurdles against an adequate view for surgeons are a narrow and tortuous nasal fossa anatomy, inadequate lighting, and abundant bleeding of the mucosa due to the rich local vascularization.

Nasal endoscopic surgery, which started in the early 70s, substantially minimized the first two hurdles mentioned above. Endoscopes can reach further, magnify the field of view, and provide angulated optics, which has enabled surgeons to reach until then inaccessible recesses.^1^ However, the third hurdle - bleeding - has in a way increased. With surgery being guided by an endoscopic image, any minor bleeding that covers the tip of the endoscope may cloud the surgeon's view. Thus, hemostasis of the nasal mucosa, which was already important, has become paramount for operability.

Except for septoplasty, in which some authors find no benefit in using vasoconstrictive solutions,^2^^,^^3^ all other surgeries use these agents for controlling blood loss. The use of cocaine and its derivatives have been widely used. However, its price and use for illicit ends has led many countries to ban these substances. Adrenalin has become popular because it is inexpensive and available in nearly every hospital.

The major difficulty with adrenalin is to establish the dose that provides the best safety and efficacy; there is no standard concentration defined for this substance in the medical literature. Furthermore, when applying adrenalin topically, the total amount that is used is hard to establish.

Adrenalin concentrations - when mentioned - have varied widely in the literature, ranging from 1:200 000 to 1:1 000 for topical use, and 1:200 000 to 1:50 000 for infiltration.^1^^,^^4^^,^^5^ The idea that higher concentrations of adrenalin are needed for improved operability in nasal endoscopic surgery has subtly gained strength. This has been due mostly to the protocols used in health care centers with more experience in endoscopic surgery worldwide, which adopt concentrations for topical use of 1:5 000, 1:2 000 or 1:1 000.^6^^,^^1^

Although there has been a world trend to use more concentrated solutions, studies demonstrating improved hemostasis and safety (incidence of systemic effects) with these concentrations, compared to lower concentrations, are lacking. Systemic side effects are proportional to the absorption of adrenalin, another topic that has been poorly investigated.

The purpose of this study was to compare the topical use of adrenalin solutions at different concentrations in nasal endoscopic surgery, assessing its efficacy in hemostasis, systemic absorption, and onset of adverse effects.

## PATIENTS AND METHODS

A prospective controlled double-blind study was done on a sample of randomly selected patients. The Research Ethics Committee of the Hospital X approved this study (approval number 207/04 - CEP).

Fifty-four consecutive patients aged over 18 years undergoing endoscopic nasal surgery for the treatment of nasosinusal polyposis at our hospital were selected.

Inclusion and exclusion criteria are shown on Table 1. Before surgery, all patients were assessed with an electrocardiogram, the prothrombin time (PT), the activated partial thromboplastin time (PTT), and a surgical risk assessment by a clinician or cardiologist. Only patients classified preoperatively as ASA class I or II (American Society of Anesthesiology) were included.^7^ Patients with signs of cardiac disease, elevated systemic blood pressure, or blood dyscrasia were excluded. Eligible patients signed a free informed consent form.

**Table 1:** Inclusion and exclusion criteria

Inclusion criteria:
1) Age equal to or over 18 years.
2) Patient with nasosinusal polyposis, for which surgery was indicated.
3) Classified preoperatively as class I (patient with no comorbidities) or class II (mild systemic disease), according to the classification system of the American Society of Anesthesiology.7
**Exclusion criteria:**
1) Patients legally incompetent or unable to provide consent.
2) Pregnant patients.
3) Patients with systemic arterial hypertension.
4) Patients with coronary disease.
5) Patients with cardiac arrhythmias or with any anomalies of the cardiac rhythm in a preoperative electrocardiogram.
6) Patients with collagen vascular diseases.
7) Patients with acute or chronic kidney or liver failure.
8) Patients with coagulation disorders or any type of blood dyscrasias.
9) Patients using sympathomimetic drugs, alpha-agonists, beta-agonists, alpha-blockers, beta-blockers, chlorpromazine, haloperidol, teophilin, aminophilin, cocaine, quinine, reserpine, imipramine, levodopa or anticoagulants.
10) Patients that did not interrupt hypoglycemiant drugs at least 48 hours before surgery or non-steroidal anti-inflammatory drugs at least 4 weeks before surgery.
11) Patients that were given other muscle relaxants, hypnotic drugs, and volatile anesthetics other than those established in the research protocol.

All patients were given prednisone (40 mg/day) during five days before surgery, followed by gradual removal postoperatively.

Patients were chosen randomly by a draw to one of three study groups, according to the concentration of adrenalin used during surgery: 1% lidocaine solution with adrenalin at 1:2 000, 1:10 000 or 1:50 000 concentrations. An anesthesiologist who was not involved in the surgery prepared the solution. Surgeons and the surgical team were unaware of which solution was used. The anesthesiologist that was involved in the surgery was also unaware of the concentration, but could request this information if it was deemed necessary at any point during surgery.

### Anesthesia

Preanesthetic sedation was not used in any patient. Induction of anesthesia was done in all patients with propofol and alfentanil. Maintenance of anesthesia was done with propofol and fentanil, repeating the doses as needed. Muscle relaxation was attained with rocuronium and/or atracurium. Controlled positive pressure ventilation (33% oxygen in nitrous oxide) was used in all cases. The inhaled anesthetic was isoflurane.

### Using the adrenalin solution

Adrenalin solutions were used topically. Cotton strips were imbibed with the solution; the excess was carefully removed until the cotton strip was saturated but not dripping even upon compression. Removal of the excess solution was done over the recipient containing the solution to avoid waste. Each strip absorbed about 1 ml of the solution. The cotton strips were then inserted in the nasal fossae (one each side) for 4 minutes in all of the surgeries.

During the operation, the surgeon could use the cotton strips again for hemostasis; the strips were always prepared in the same manner. Each surgery used 20 cotton strips. After the first use, each cotton strip was kept in contact with the nasal fossa for 2 minutes. In reuse, each strip was placed in the nasal fossa that was not being operated on at that particular moment, while the surgeon operated on the other side, to avoid increasing the surgical time. In all cases at the end of surgery, 20 ml of the topical solution had been used in the cotton strips, which were kept in contact with the nasal mucosa during 40 minutes in each case. Thus, the total quantity of adrenalin that was given was 400 μg in the 1:50 000 solution, 2 000 μg (2 mg) in the 1:10 000 solution, and 10 000 μg (10 mg) in the 1:2 000 solution.

The surgeries were carried out by the same surgeons (the main researcher). The electrocautery was used parsimoniously to minimize damage to the nasal mucosa and to avoid prolonged postoperative healing.

### Assessing the extent of disease

The extent of nasosinusal polyposis was estimated by the number of anatomical structures that were operated in each surgery (inferior turbinate, middle turbinate, ethmoid bulla, unciform process, posterior ethmoid, sphenoid, and frontal recess, counted for each nasal fossa), based on the principle that the number of structures that need operating is correlated with the extent of disease. We used the term procedure to describe the operation on each anatomical structure. Thus, the number of procedures in each surgery was recorded.

### Assessing perioperative bleeding

Three forms of evaluating bleeding were used: volume of aspirated blood, the surgeon's grade of bleeding, and an assessment of bleeding based on a visual-analog scale by the surgeon.

At the beginning of each surgery, gauze was placed on the rhinopharynx to stop blood from reaching the pharynx. The volume of saline used during surgery was measured. At the end of surgery, the total amount of aspirated blood was measured. Blood absorbed by the gauze or cotton strips with adrenalin was not included, as was not any blood that the patient swallowed.

At the end of surgery, the surgeon classified perioperative subjectively; this was done grading the amount of bleeding from “A” to “D” as shown on Table 2, and using a visual-analog scale to score the amount of bleeding from “no bleeding” to “extreme bleeding”. The distance from the left tip of the scale until the grade given by the surgeon was measured to one decimal point, which resulted in a 0 to 10 score for bleeding.

**Table 2:** Scores of perioperative bleeding.

Score A	Minimal bleeding.
Score B	Controllable bleeding; did not affect the surgery as planned.
Score C	Increased bleeding, affecting the surgery but not impeding its planned outcome using endoscopy.
Score D	Extreme bleeding, that did not allow the procedure to be concluded as planned, or that required a change to a non-endoscopic surgery.

### Dosing plasma catecholamines

Plasma catecholamines (adrenalin and noradrenalin) were dosed three times in each patient. The first sample was taken 10 to 15 minutes after induction of anesthesia, before any surgical stimulation or use of vasoconstrictors. The second sample was taken after using the tenth cotton strip with adrenalin, and the third sample was collected after having used the last cotton strip with adrenalin, which roughly coincided with the end of surgery.

Tubes containing ethylenediaminetetraacetic acid (EDTA-K3) and a 10 ml syringe (both cooled to −4°C prior to collecting the sample) were used. The samples at −4°C were immediately centrifuged at 3 000 rotations per minute during 10 minutes. Next, plasma was separated and stored at −80°C until the moment of analysis.

Plasma catecholamines were measured by liquid chromatography with electrochemical detection. This technique was meticulously described by Causon et al. in 1981.^8^

Normal limits for the analyses above are adrenalin ≤ 115 pg/ml and noradrenalin = 70 to 750 pg/ml.

### Recordings of the heart rate, blood pressure and heart rhythm

The arterial blood pressure was measured and recorded automatically every three minutes between the point when the first and third blood sample were collected. The heart rate was measured continuously on a cardioscope. The heart rate measured jointly with the arterial blood pressure every three minutes were used for further analysis. At any point if the heart rate was above 115 beats per minute or less than 55 beats per minute, this value was also recorded. The heart rhythm was monitored throughout surgery to check for arrhythmias.

Normal and elevated heart rate and arterial systolic and diastolic pressures were defined according to the Seventh Report of the Joint National Committee on the Prevention, Detection, Evaluation, and Treatment of High Blood Pressure.^9^^,^^10^

### Control group

As it was not possible to have a control group of patients undergoing nasal endoscopic surgery without vasoconstrictors, a control group was established containing patients undergoing tonsillectomy under general anesthesia with the same anesthesiological protocol without the use of topical or infiltrated adrenalin. Three blood samples were taken from each patient during surgery to measure plasma catecholamines; additionally, perioperative hemodynamic parameters were measured.

The control group was also used for comparing the cardiovascular parameters and the levels of plasma catecholamines. Comparisons of surgical times and perioperative bleeding were made among the three study groups that underwent nasal endoscopic surgery, since there was no sense in comparing these parameters among different surgeries.

### Data analysis

A multivariate analysis was made to verify whether there was any statistically significant difference in the results among the groups; additional analyses included a comparison of the sex distribution, age, and severity of disease. The one-way analysis of variance, or the non-parametric Kruskal-Wallis ANOVA was used for the comparison among three (or four) groups; some variables did not have a normal (Gaussian) distribution.

The multiple comparison Tukey test, or the corresponding non-parametric test based on the Kruskal-Wallis statistics, was applied to identify the groups that differed among themselves.^11^ The Dunnet multiple comparisons test was applied to identify the experimental groups that differed from the control group. The chi-square (c^2^) test or Fisher's exact test were used for comparing qualitative variables among the groups. The repeated measures ANOVA was applied to analyze the behavior of measurements across time (three or six assessments) for each group separately. The Bonferroni multiple comparisons test (adjusted for repeated measures) was applied to identify the moments that differed. The one factor repeated measures ANOVA was applied to verify whether behavior across time differed among the groups.

The significance level was 5%.

## RESULTS

Five of 54 patients who signed the free informed consent form were eventually excluded, 2 because the adrenalin solution was applied inadvertently in the nasal fossae before the first blood sample had been taken, and 3 because the blood samples were handled inadequately. Thus, 49 patients were left for the analyses; these patients were allocated randomly to the three groups. The control group consisted of 10 patients.

There was no sex or age difference among groups; the age ranged from 18 to 54 years (mean – 34.2 years). The histopathological diagnosis of nasosinusal polyposis was confirmed for all patients in the study groups.

There was no statistically significant difference among the three groups as to the procedures (anatomical structures that were operated); this led us to assume that there was a homogeneous distribution of the severity of disease among groups. The mean was 10.6 procedures per surgery.

### Operative time

The mean operative time was 1602 minutes in the adrenalin 1:2 000 group, 171.5 minutes in the adrenalin 1:10 000 group, and 190.1 minutes in the adrenalin 1:50 000 group. Although different, these values were not statistically significant (p value = 0.075). This was because the operative time varied widely in each group.

To overcome this difficulty, the analysis of operative time was made based on each procedure, obtained by dividing the operative time of each surgery by the number of procedures done in each. The operative time by procedure was 16.1 minutes in the adrenalin 1:2 000 group, 18 minutes in the adrenalin 1:10 000 group, and 19.1 minutes in the adrenalin 1:50 000 group. These differences were statistically significant (p value = 0.02), showing that surgery using adrenalin 1:2 000 took less time.

### Bleeding

Table 3 shows the, standard deviation (SD), median, minimum and maximum subjective scores of bleeding per group, the volume of aspirated blood, and the corresponding descriptive level in the statistical test (p value).

**Table 3:** Statistical analysis of perioperative bleeding variables (in milliliters) for each group.

Group	N	Mean	D.P.	Median	Minimum	Maximum	p value^a^	Significant differences^b^
Adrenalin 1:2000	17	140,3	56,7	135	40	270	0,0001	
Adrenalin 1:10.000	16	336,9	20,40	315	85	750		1:2000 ≠ 1:10.000 1:2000 ≠ 1:50.000 1:10.000 ≠ 1:50.000
Adrenalin 1:50.000	16	425,8	25,58	334	100	1100		

SD: Standard deviation p value^a^: Kruskal-Wallis ANOVA for the three study groups.

Sig. dif.^b^: Tukey's multiple comparisons test at 5%.

The mean bleeding was 140.3 ml in the group in which adrenalin 1:2 000 was used, which was 2.4 times less than bleeding in the adrenalin 1:10 000 group (mean – 336.9 ml), and three times less that bleeding in the adrenalin 1:50 000 group (mean – 425.8 ml).

In the subjective assessment of bleeding, only two patients were graded “D” - “extreme bleeding” - which did not allow the procedure to continue as planned or that required a change to a non-endoscopic technique. Both patients belonged to the adrenalin 1:50 000 group. In one of these cases, bleeding interfered with the opening of the sphenoid sinus (as planned beforehand); in the other cases, the endoscope had to be left aside, and surgery continued with the naked eye and a nasal speculum until adequate control of bleeding was attained and the endoscope was reapplied. Since only two patients scored “D” for bleeding, these were considered as a single group (scores “C” and “D”) in the statistical analysis.

**Chart 1:**
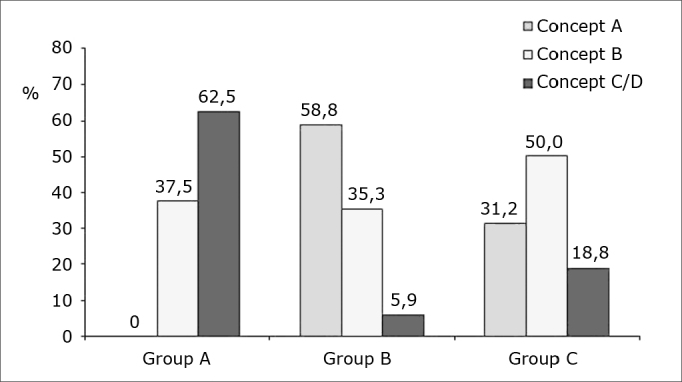
Distribution of intraoperative bleeding concepts (in percentage) per group.

The topical adrenalin 1:50 000 group had a significantly higher C/D scores (62.5%) compared to the adrenalin 1:2 000 group (5.9%) and the adrenalin 1:10 000 group (18.8%). On the other hand, the adrenalin 1:2 000 and 1:10 000 groups had a higher rate of scores A (58.8% and 31.2%) compared with the adrenalin 1:50 000 group (0%); these findings are shown on Chart 1. These differences were statistically significant (p value = 0.0002).

In the subjective visual-analog scale for bleeding, the score in the adrenalin 1:2 000 group (mean – 1.5 ± 0.8 SD) was lower compared to the adrenalin 1:10 000 group (mean – 3.3 ± 1.2 SD), which in turn scored lower than the adrenalin 1:50 000 group (mean – 5.3 ±1.2 SD). These differences were statistically significant (p value = 0.0001).

### Dosage of plasma catecholamines

**Chart 2:**
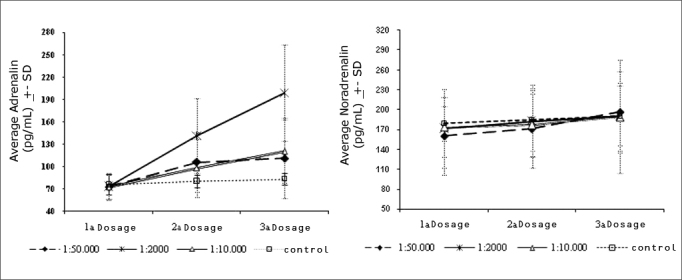
Variations of adrenalin and noradrenalin dose averages throughout the operation for each group. DP=Standard deviation.

Chart 2 shows the variation of plasma adrenalin and noradrenalin levels in the three study groups and the control group. Plasma adrenalin levels increased gradually as surgery progressed (and in all three measurements) in all groups except in the control group (p values = 0.0001; 0.0001 and 0.00^13^ for the adrenalin 1:2 000, 1:10 000 and 1:50 000 groups). The one factor repeated measures ANOVA revealed that the increase was higher in the adrenalin 1:2 000 group compared to the other groups (p value = 0.0001). In this group, the mean plasma concentration of adrenalin reached 198.6pg/ml (a 192% increase relative to baseline values, and 1.7 times higher than the normal upper limit); the highest recorded peak was 346pg/ml (over three times the maximum normal limit, and a 409% increase compared to baseline levels). The highest mean measure (3rd dosage) in the adrenalin 1:10 000 group was 120.6pg/ml; the highest recorded peak was 265pg/ml. The highest peak in the adrenalin 1:50 000 group was 111.4pg/ml (a 64% increase compared to baseline levels, but still within normal limits); the highest recorded peak was 217pg/ml.

Noradrenalin concentrations did not vary significantly in any of the three measurements in all groups. Thus, the adrenalin/noradrenalin ratio increased in all groups as surgery progressed, except in the control group.

### Cardiac and cardiovascular hemodynamic parameters

No arrhythmias were detected in any patient during perioperative cardiac monitoring.

A comparison of the mean and median heart rates (HR), systolic arterial pressure (SAP) and diastolic arterial pressure (DAP) in each group at any given moment showed that these measures were within normal limits in all groups.

The occurrences of SAP and DAP above normal limits (hypertension peaks) revealed a higher number of such occurrences in the more concentrated adrenalin groups (1:2 000 and 1:10 000), as shown on Table 4. There were 53 SAP hypertensive peaks (values ≥ 140 mmHg) in the adrenalin 1:2 000 group, and 62 DAP hypertensive peaks (values ≥ 90 mmHg); there were similar findings in the adrenalin 1:10 000 group (59 SAP peaks and 48 DAP peaks). The occurrences of blood pressure peaks in the adrenalin 1:50 000 group were only 5 SAP peaks and 20 DAP peaks. In the control group, in which adrenalin was not given, there were 8 SAP measures above normal and only 1 DAP measure above normal. The difference in the occurrence of hypertensive peaks in the adrenalin 1:2 000 and 1:10 000 groups compared to the other groups was statistically significant (p = 0.0001). There were no HR measures or tachycardia significantly above normal in any group.

**Table 4:** Distribution of the measured hemodynamic parameters as normal and abnormal values for each group.

Parameter	Values	Adrenalin 1:2000	Adrenalin 1:10.000	Adrenalin 1:50.000	Group Control	p value^a^	Significant differences^b^
	< 100 bpm	912 (99,2%)	915 (99,9%)	1013 (99,6%)	236 (99,6%)		
HR		7 (0,8%)	1 (0,1%)	4 (0,4%)	1 (0,4%)	0,45	None
		0	0	0	0		
	< 140 mmHg	866 (94,2%)	857 (93,6%)	1012 (99,5%)	229 (96,6%)		140˜159 mmHg at 1:2 000 and 1:10 000 ≠ 1:50 000 and Control
SAP						< 0,0001	
		40 (4,3%)	48 (5,2%)	5 (0,5%)	8 (3,4%)		160˜179
		11 (1,3%)	10 (1,1%)	0	0		mmHg at 1:2 000 and 1:10
		2 (0,2%)	1 (0,1%)	0	0		000 ≠ 1:50 000
	< 90 mmHg	857 (93,3%)	868 (94,8%)	997 (98%)	235 (99,2%)		
	90 ˜ 99 mmHg	52 (5,7%)	45 (4,9%)	18 (1,8%)	1 (0,4%)		90˜99 mmHg at 1:2 000 and 1:10 000 ≠ 1:50 000 and Control
DAP	100 ˜ 109 mmHg	8 (0,9%)	3 (0,3%)	2 (0,2%)	1 (0,4%)	< 0,0001	
	> or equal to 110 mmHg	2 (0,1%)	0	0	0		

HR = Heart rate SAP = Systolic arterial pressure DAP = Diastolic arterial pressure

MAP = Mean Arterial Pressure p valuea: repeated measures ANOVA

Sig. dif.b: Bonferroni multiple comparisons

We then attempted to verify it these hypertensive peaks were sudden or acute variations of the arterial pressure immediately after applying cotton strips with the adrenalin solution. We correlated the placement of cotton strips with arterial blood pressure measurements taken within 10 minutes of applying topically each cotton strip with adrenalin. No correlation was established between hypertensive peaks and such 10-minute periods.

For the next analysis, occurrences of arterial pressure above normal were divided into two groups: peaks in the first half of surgery, and peaks in the second half of surgery. Hypertensive peaks were at least twice as frequent in the second half of surgery compared to the first half of surgery in the more concentrated adrenalin groups (1:2 000 and 1:10 000), as shown on Table 5. This finding suggests that pressure levels tend to become more altered as surgery progresses.

**Table 5:** Distribution of the measured hemodynamic parameters above normal values in each group in relation to operative time.

Parameter	Occurrences	Adrenalin concentration	First half of operative time	Second half of operative time	p value^a^	Significant differences^b^
HR (≥ 100 bpm)	4	1:2000	5	2		
	7	1:10.000	1	–		
	1	1:50.000	1	3	0,71	None
	1	Controle	1	–		
		5	1:2000	12	41	Groups B and C had more occurrences in the second half compared to group A and the Control.
SAP (≥ 140 mmHg)	53	1:10.000	18	41	0,042	
	59	1:50.000	2	3		
	8	Control	3	5		
	20	Group B (1:2000)	21	41		
DAP (≥ 90 mmHg)	62	Group C (1:10.000)	14	34		Groups B and C had more occurrences in the second half compared to group A and the Control.
				< 0,05		
	48	Group A (1:50.000)	12		8	
	2	Control	2		–	

HR = Heart rate SAP = Systolic arterial pressure DAP = Diastolic arterial pressure

MAP = Mean Arterial Pressure p valuea: repeated measures ANOVA

Sig. dif.b: Bonferroni multiple comparisons

**Chart 3:**
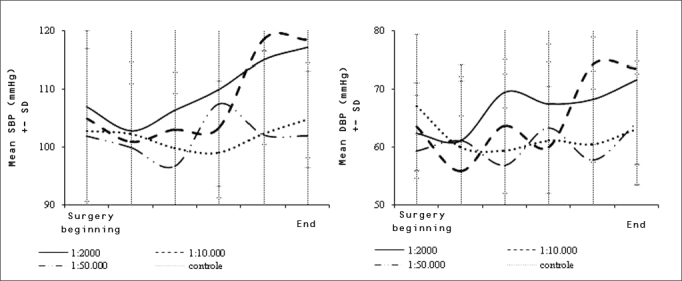
Variations in systolic blood pressure (SBP) and diastolic blood pressure (DBP) throughout the operation for each group. DP=Standard Deviation.

The behavior of SAP and DAP as surgery progressed showed that these values increased gradually in the adrenalin 1:2 000 and 1:10 000 groups, which was statistically significant (p values = 0.028 and 0.0001 - Chart 3). Even with such changes, the mean SAP and DAP measurements at the end of surgery remained within normal limits in all groups, as mentioned above. This was not the case with the HR.

## DISCUSSION

Few authors have investigated the effects of vasoconstrictors on the nasal mucosa. Comparing the results of published papers is difficult, since each deals with different surgical procedures (septoplasty,^2^^,^^3^^,^^6^ turbinectomy,^5^ endoscopic surgery of the lateral wall of the nose^4^^,^^12^), a variety of administration methods (submucosa, topical or both^4^^,^^6^^,^^12^^,^^13^^,^^14^), and vasoconstritors (cocaine, adrenalin, phenylephrin,^15^ oxymetazoline^12^). Often these studies tend to associate more than one vasoconstritor, usually adrenaline and cocaine.^14^^,^^16^ In our view, not enough information is available about the behavior of each vasoconstritor singly on the nasal mucosa; thus, such associations are not beneficial for research purposes, since they complicate data analysis.

This is the first paper that we know of comparing adrenalin solutions (not associated with other vasoconstritors) used only topically on the nasal mucosa.

We found the difference in bleeding in the three groups extremely significant. In the absence of demonstrated toxicity, a 2.4 higher effectiveness of the adrenalin 1:2 000 solution compared with the 1:10 000 solution would without any doubt justify its use. But what could be said about the toxicity of this drug at various concentrations for topical use?

First of all, we may begin with the principle that the most important adverse effects depend on the systemic absorption of adrenalin following topical use.

It is not enough to show increased plasma levels of adrenalin to study systemic absorption. Adrenalin (or epinephrine) is not a synthetic substance; it is produced by the adrenal gland, which may increase its production in response to stressful conditions, including the stress of surgery. Thus, there is the need to differentiate an increased endogenous production from a possible exogenous absorption.

We used two methods to achieve this end. The first one is based on the principle that increased endogenous production of adrenalin is always accompanied by an increased sympathetic tonus and elevated plasma levels of noradrenalin.^17^ Our findings showed that elevated plasma concentrations of adrenalin were not followed by increased noradrenalin levels; this suggests that increased plasma adrenalin levels were due to exogenous absorption of this substance. The second method made use of the control group, which was subjected to surgical stress, a similar anesthesiological procedure, and no vasoconstritor. Plasma adrenalin levels did not vary in the control group; this, then, is further evidence that there was increased exogenous absorption in the adrenalin groups.

We found a gradual increase in the plasma levels of adrenalin in all three groups in which it was used; these levels were higher in the adrenalin 1:2 000 group. In these three groups, however, such increased levels were lower than those found when adrenalin is infiltrated in the nasal submucosa.^6^^,^^18^^,^^19^ When this approach was used, plasma adrenalin levels were up to 40 times higher compared to baseline levels (over 3 000 pg/ml); in out study, peak levels were 2 to 3 times higher than baseline values (the highest plasma adrenalin level was 346 pg/ml). This is evidence of a probably difference in adrenalin absorption when applied by these two routes. In the slower topical absorption, rapid adrenalin metabolism comes into play (its half life is only two minutes), and peak concentrations are avoided.

It is true that our measurements were made throughout the surgery; eventual sudden variations immediately after each cotton strip was applied may not have been measured. However, behavior of hemodynamic parameters did not vary suddenly after each cotton strip was used, but increased slowly and progressively, suggesting that higher peaks than those we recorded did not occur. Furthermore, van Hasselt et al., in a comparison of topical and submucosal adrenalin use (associated with 10% cocaine), had already reached similar conclusions about differences in absorption and peak levels between both administration routes.^6^

Since cotton strips with adrenalin were maintained in place for about the same time (40 minutes) in all patients, if we divide the mean plasma adrenalin increase in each group by that time, we end up with an elevation rate per minute of topical solution use. This rate of increase was 3.13pg/ml per minute of use of cotton strips in the adrenalin 1:2 000 group, 1.20 pg/ml per minute in the adrenalin 1:10 000 group, and 0.95pg/ml per minute in the adrenalin 1:50 000 group.

Maximum values in each group (346pg/ml in the adrenalin 1:2 000 group, 265 pg/ml in the adrenalin 1:10 000 group, and 217pg/ml in the adrenalin 1:50 000 group) are close to those attained occasionally by the body under specific conditions. For instance, intense physical exercise may raise plasma adrenalin levels to about 350pg/ml without any threat to individuals.^20^ It is, in fact, a beneficial adaptation of the body to that condition. Thus, it could be said that the peak levels in our sample are not truly harmful. Differences could arise depending on the time that such levels remained above normal; in surgeries, this time was not short.

No patient in our sample manifested cardiac arrhythmias. Arrhythmias associated with adrenalin were common when halothane used to be used as an anesthetic, since this drug increases the sensitivity of adrenalin myocardial adrenergic receptors. With further developments in anesthesia, new drugs now provide more hemodynamic stability and interact less with adrenalin, making adrenalin-related arrhythmias rare.

Similar to plasma adrenalin concentrations, blood pressure levels in the more concentrated adrenalin groups (1:2 000 and 1:10 000) increased slowly and gradually as surgery progressed, which suggests a possible correlation between both findings. This did not occur in the control group and the adrenalin 1:50 000 group (in the latter blood pressures rose slightly, but there was no statistical significance).

If we divide the mean increase in SAP and DAP levels by the total duration of exposure to the topical adrenalin solution, the result is a 0.50 mmHg (SAP) and a 0.37 (DAP) mmHg increase rate for each minute of topical adrenalin use at 1:2 000. At a concentration of 1:10 000, the rate is 0.34 mmHg (SAP) and 0.25 mmHg per minute of cotton strip use.

Although mean SAP and DAP values were within normal limits in all groups even close to the end of surgery, hypertensive peaks were more frequent in the groups that were given more concentrated adrenalin solutions.

Our anesthesia protocol consisted of inhaled and venous anesthesia combined. Some papers have suggested that venous anesthesia is superior for maintaining hemodynamic stability, which might have avoided the hypertensive peaks we encountered.^21^^,^^22^ Anderhuber et al. studied 51 patients that were given a topical adrenalin 1:1 000 solution associated with submucosal adrenalin 1:200 000 (in patients under fully venous general anesthesia), and found no increases in blood pressure during surgery.^4^ Additional controlled studies, preferably with larger series and more homogeneous groups, are needed for more definitive conclusions.

## CONCLUSION

This study clearly demonstrates the haemostatic benefit of adrenalin at 1:2 000. In terms of adverse effects, there was a tendency for the systemic arterial pressure to increase as more concentrated solutions were used, although mean supraphysiological levels were not reached. Concerning this point, the topic remains controversial; further studies are thus needed.
